# Characteristics of the Diploid, Triploid, and Tetraploid Versions of a Cannabigerol-Dominant F_1_ Hybrid Industrial Hemp Cultivar, *Cannabis sativa* ‘Stem Cell CBG’

**DOI:** 10.3390/genes12060923

**Published:** 2021-06-17

**Authors:** Seth Crawford, Brendan M. Rojas, Eric Crawford, Matthew Otten, Thecla A. Schoenenberger, Andrea R. Garfinkel, Hsuan Chen

**Affiliations:** 1 Oregon CBD, Independence, OR 97351, USA; seth@jackhempicine.com (S.C.); brendan@jackhempicine.com (B.M.R.); eric@jackhempicine.com (E.C.); mjotten@jackhempicine.com (M.O.); thecla@jackhempicine.com (T.A.S.); andrea@jackhempicine.com (A.R.G.); 2 Department of Horticultural Science, North Carolina State University, Raleigh, NC 27695, USA

**Keywords:** polyploidy, ploidy manipulation, infertility, asymmetric compatibility, inter-ploid compatibility, asymmetric triploid block, hemp breeding

## Abstract

Hemp (*Cannabis sativa* L.) has recently become an important crop due to the growing market demands for products containing cannabinoids. Unintended cross-pollination of *C. sativa* crops is one of the most important threats to cannabinoid production and has been shown to reduce cannabinoid yield. Ploidy manipulation has been used in other crops to improve agronomic traits and reduce fertility; however, little is known about the performance of *C. sativa* polyploids. In this study, colchicine was applied to two proprietary, inbred diploid *C. sativa* inbred lines, ‘TS1-3’ and ‘P163’, to produce the tetraploids ‘TS1-3 (4*x*)’ and ‘P163 (4*x*)’. The diploid, triploid, and tetraploid F_1_ hybrids from ‘TS1-3’ × ‘P163’, ‘TS1-3 (4*x*)’ × ‘P163’, and ‘TS1-3 (4*x*)’ × ‘P163 (4*x*)’ were produced to test their fertilities, crossing compatibilities, and yields. The results indicated a reduction in fertility in the triploids and the tetraploids, relative to their diploid counterparts. When triploids were used as females, seed yields were less than 2% compared to when diploids were used as females; thus, triploids were determined to be female infertile. The triploids resulting from the crosses made herein displayed increases in biomass and inflorescence weight compared to the diploids created from the same parents in a field setting. Statistical increases in cannabinoid concentrations were not observed. Lastly, asymmetric crossing compatibility was observed between the diploids and the tetraploids of the genotypes tested. The results demonstrate the potential benefits of triploid *C. sativa* cultivars in commercial agriculture.

## 1. Introduction

*Cannabis sativa* L. is an annual row crop grown for protein, oil-rich grain, multiple industrial usage fibers, and for the secondary metabolites produced by the plant (referred to as “cannabinoids”) for medicinal and recreational use [1,2]. Most *C. sativa* plants display a dioecious habit, whereby male and female organs develop on different plants [3], a trait regulated by an XY chromosome sex determination system [4]. While for grain and fiber *C. sativa* production, both male and female plants are grown and valued, but only female plants are desired for the medicinal and recreational cultivation of cannabinoids [3], which are produced at high concentrations in the glandular trichomes located on the bracts of female flowers [5].

Avoiding pollination of female flowers during the production of *C. sativa* for cannabinoids is a priority for growers of this crop, as pollination has been shown to reduce essential oil yield by more than 55% [6]. Furthermore, in the trimmed flower market, the presence of seeds is undesirable for the consumer. Growing only genetically female plants is the most common strategy currently used to avoid accidental pollination. Although molecular tests are available to identify genetic males prior to transplanting in the field [7], using either “feminized seed” (seeds that are produced using two genetic females) or vegetatively propagated females are the current industry standards. Despite the ease with which genetic females can be produced, the occasional production of male flowers on genetically female *C. sativa* plants (referred to as “hermaphrodism”) [8] still necessitates diligent scouting of fields and the removal of pollen-bearing plants for successful, essential oil hemp production.

Pollen drift from outside a managed field presents another challenge for *C. sativa* growers. *C. sativa* is a wind-pollinated plant that produces large numbers of pollen grains, easily spread over distances of up to 300 km [3]. Drifting *C. sativa* pollen could come from multiple sources, including poorly managed medicinal and recreational *C. sativa* farms, fiber and grain hemp farms where male plants are encouraged, or from escaped or naturalized *C. sativa* plants (sometimes referred to as “feral” *Cannabis*). Preventing pollination by drifting pollen can be exceedingly difficult––if not impossible––for growers, and therefore represents a severe limitation to where *C. sativa* can be successfully cultivated.

The breeding of triploid cultivars has been used as a strategy to produce non-invasive or infertile cultivars in many crops, including *Acer* spp. [9], *Humulus lupulus* [10], *Hypericum androsaemum* [11], and *Miscanthus sinensis* [12]. Reports have speculated that triploid *C. sativa* cultivars may be infertile, and therefore resistant to the yield damage caused by pollination [13]; however, we were unable to locate studies demonstrating triploid hemp sterility.

Several other advantages to ploidy manipulation of crop species have been documented in the literature. Specifically, ploidy manipulation has been shown to increase secondary metabolite yields in several medicinally important or essential oil crops, such as *Papaver bracteatum* [14], *Lavandula vera* [15], *Echinacea purpurea* [16], and *C. sativa’s* closest relative of economic importance, *Humulus lupulus* [10]. Furthermore, polyploid versions of several ornamental plant species, such as the interspecific hybrids of poinsettia (*Euphorbia pulcherrima* × *E. cornastra*) [17], and monk verbena (*Glandularia peruviana* × *G. scrobiculata*) [18], and hybrids of myrtle (*Lagerstroemia indica*) [19], impatiens (*Impatiens balsamina*) [20,21], and hibiscus (*Hibiscus rosa-sinensis*) [22], have been shown to display significantly larger flowers than their diploid counterparts.

Several ploidy manipulations in *C. sativa* have been reported in the literature [13,23,24]; however, knowledge of the effects on chemical profiles is limited to the differences between tetraploid and diploid type I and type II plants, those that are tetrahydrocannabinol (THC)-dominant, or those that produce both THC and cannabidiol (CBD), respectively. These studies indicated that although THC concentration remained unchanged in polyploids, CBD production was slightly increased [23,24]. A method of producing triploid type III plants, those that are CBD-dominant, has also been reported [13]; however, the field phenotype and fertility status of the triploid plants were not discussed. Furthermore, we were unable to locate any studies discussing the impact of ploidy manipulation on type IV, cannabigerol (CBG)-dominant, cultivars. Given the potential advantages of ploidy manipulation in *C. sativa*, our goal was to produce and test the fertility and cannabinoid content of diploid, triploid, and tetraploid type IV *C. sativa* plants.

In this study, a colchicine treatment was used to produce tetraploid versions of two CBG-dominant inbred lines, ‘TS1-3’ and ‘P163’. Diploid (2*x*), triploid (3*x*), and tetraploid (4*x*) F_1_ hybrid seeds were produced from crossing the combinations TS1-3 (2*x*) × P163 (2*x*), TS1-3 (4*x*) × P163 (2*x*), and TS1-3 (4*x*) × P163 (4*x*). Several crosses between these F_1_ hybrids were attempted to determine intraploid compatibilities. The seed number produced by each pollinated plant was counted. The cannabinoid yield, dry biomass, inflorescence weight, and cannabinoid concentrations of different ploidy hybrid plants, grown both indoors and outdoors, were also measured.

## 2. Materials and Methods

### 2.1. Plant Material

*C. sativa* inbred lines ‘TS1-3’ and ‘P163’ were used as parents in all experiments; both are female inbred lines developed by Oregon CBD (Independence, Oregon). Additional information on ‘TS1-3’ is available in Garfinkel et al. [25]. Diploid F_1_ hybrid seed of these parents has been released as ‘Stem Cell CBG’ and triploid F_1_ hybrid seed has been released as ‘Stem Cell CBG Seedless’. In this study, the ‘TS1-3’ inbred line was clonally propagated whereas ‘P163’, a day-length neutral inbred line, was propagated by seeds produced from the selfing of ‘P163’.

Tetraploid versions of the ‘TS1-3’ and ‘P163’ lines were produced by different methods. Tetraploid ‘TS1-3’ was induced by a modified method of in vitro induction [24] by a 36-h 0.25% colchicine treatment. The colchicine-treated ‘TS1-3’ plants were then grown in a greenhouse and clonally propagated for three generations. Twenty plantlets were colchicine-treated, resulting in five tetraploid clones. Three of the tetraploid plants remained stable over three generations of asexual propagation. No phenotypical differences were observed among the three clones; therefore, a single clone was randomly selected to serve as the tetraploid parent for trials, hereafter referred to as ‘TS1-3 (4*x*)’. Tetraploid versions of ‘P163’ were produced by treating young seedlings for 48 h with a 0.2% colchicine solution [23]. Colchicine treatments were applied to the apical meristem of forty ‘P163’ seedlings immediately after cotyledons opened. Six colchicine-treated seedlings that were estimated to be tetraploids were then treated with silver thiosulfate (STS, see below) to produce selfed seeds. Twenty seeds from each of the six families were sown and their ploidies were tested. The resultant tetraploid ‘P163’ plants will hereafter be referred to as ‘P163 (4*x*)’. Pollen from two randomly chosen ‘P163 (4*x*)’ plants were bulked and used in pollination trials. The ploidy of all colchicine-treated plants and seedlings were estimated by flow cytometry.

**Figure 1:**
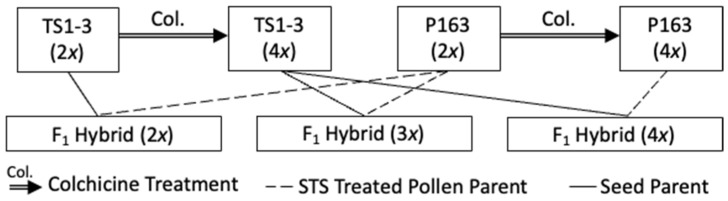
Crossing combinations used to produce diploid, triploid, and tetraploid F_1_ hybrid *C. sativa* plants. Colchicine treatments were applied on commercial inbred lines ‘TS1-3’ and ‘P163’ to produce tetraploid versions. The silver thiosulfate (STS) treatment was applied to ‘P163’ and ‘P163 (4*x*)’ plants to produce viable pollen. The diploid, triploid, and tetraploid F_1_ hybrids were made from crossing combinations of TS1-3 (2*x*) × P163 (2*x*), TS1-3 (4*x*) × P163 (2*x*), and TS1-3 (4*x*) × P163 (4*x*).

All plant material directly involved in the subsequently described trials was started from seed. Seeds representing three ploidies of F_1_ hybrid plants, including diploid (*2x*), triploid (3*x*), and tetraploid (4*x*), were developed from several crossing combinations utilizing ‘TS1-3’ × ‘P163’, ‘TS1-3 (4*x*)’ × ‘P163’ and ‘TS1-3 (4*x*)’ × ‘P163 (4*x*)’ (Figure 1). Throughout this paper, the first parent indicated in the cross is the pollen recipient and the second parent reported is the pollen donor. To perform crosses, a modified silver thiosulfate (STS) treatment [26] was used to stimulate male flower development on the pollen donors, which were genetic females. The STS buffer was applied three times, once every five days, with the first treatment occurring when a solitary calyx could be observed on the axillary bud.

‘TS1-3’ (all ploidies) was not used as the pollen parent in any of the experiments as ‘TS1-3’ does not produce any viable pollen following STS treatment; this phenomenon is common in highly inbred *C. sativa* lines such as ‘TS1-3’. ‘TS1-3’ was clonally propagated for this reason as well. ‘P163’ is a day neutral selfing line that cannot be clonally propagated; therefore, seed was used.

### 2.2. Fertility and Compatibility Experiments

Inter- and intra-ploidy compatibility and fertility were assessed for the crossing combinations outlined in Figure 1. Experiments were run simultaneously in two separate growing environments to ensure there was no pollen cross-contamination. In each growing environment, eight replicates of each ploidy of F_1_ hybrid seedlings (2*x*, 3*x*, and 4*x*) were randomized, grown in 10-gallon pots, and pollinated by seven STS-treated donors that were either (1) diploid F_1_ hybrid plants, or (2) tetraploid F_1_ hybrid plants. The crossing combinations are further described in Table 1.

All plants used in the experiment were grown under 24 h of light for the first 12 weeks of development and then changed to a 12/12 light/dark cycle to stimulate flower development. Beginning at 4 weeks following the change in photoperiod, flowers on both female and converted female plants were checked daily. Male flowers were physically manipulated to release pollen and hand-held fans were used to ensure pollen dispersal; pollination occurred over a three-week period. The pollinated plants were harvested three weeks following the end of pollination to allow for seed maturation. Irrigation was cut off a week before harvest to allow the plants to dry before harvest. Each plant was harvested individually into paper bags, at which point samples were further dried at 35 °C for 7 days. The seeds of each plant were then counted. Average seed counts per plant were compared by a one-way analysis of variance (ANOVA) and post hoc means separations were conducted using Tukey’s test with a 99% confidence interval. Weights of 100 seeds from three randomly chosen plants (of those that produced at least 100 seeds) from four crossing combinations (diploid × diploid, diploid × tetraploid, tetraploid × diploid, and tetraploid × tetraploid) were taken. Seeds were determined to be “filled” or “empty” (see additional description in results) based on a visual assessment; those that were empty were easily broken. Average weights of 100 seeds were compared using a one-way ANOVA and Tukey’s separation of means test. Seeds were randomly selected, resulting in seed weights that included both filled and empty seeds.

### 2.3. Indoor Phenotyping Trials

In an independent growing environment similar to that used in the fertility tests, seven replicates of each ploidy of F_1_ hybrid seedlings (2*x*, 3*x*, and 4*x*) were grown. Plants used in the experiment were grown under 24 h of light for the first 12 weeks of development, and then changed to a 12/12 light/dark cycle to stimulate female flower development. Plants were harvested 7 weeks after the change in photoperiod in order to further assess several phenotypic traits. Plants were assessed for total plant biomass, inflorescence weight, and cannabinoid concentration. Following drying at 35 °C for 7 days, whole-plant dry weights and inflorescence weights (not trimmed to remove small leaves within the inflorescences) for each plant were weighed separately.

Analyses of cannabinoid content of the inflorescences were performed according to a modified protocol described by Vaclavik et al. [27]; details regarding the modifications can be found in Garfinkel et al. [25]. Five to 10 randomly chosen dried inflorescences from each plant were collected and used in the analysis. A 7-point calibration of combined cannabinoid standards was performed, and sample results were quantitated from integrated chromatograms.

Total cannabinoids were calculated as the sum of the acid (e.g., cannabigerolic acid, CBGA) and decarboxylated forms (e.g., CBG) of each cannabinoid detected in the sample. Combined CBGA + CBG yield is hereafter written as CBG(A). Cannabinoid yield of each plant was then calculated according to the following formula integrating flower tissue weight:

cannabinoid yield = total CBG(A) × flower tissue weight(1)

Due to unequal variances in the data, total biomass, inflorescence weight, and cannabinoid concentration, averages were compared by non-parametric methods, including a Kruskal–Wallis H test and pairwise Wilcoxon rank sum tests with a 90% confidence interval.

### 2.4. Field Trials

A total of 30 F_1_ hybrid plants, including 15 diploid and 15 triploid plants, were selected for phenotyping at a research field located in Independence, OR (44.885705° N, 123.231775° W). Diploid and triploid F_1_ hybrid seeds were sown in a seedling tray on 18 May 2020. Seedlings were transplanted into the research field at Oregon CBD (Independence, OR, USA) on 1 June 2020. Raised beds covered in black plastic mulch were constructed 6 feet apart and seedlings were hand-planted on 4-foot centers. Plants were harvested on 23 September 2020, 42 days after the appearance of the first flowers on the terminal shoot. Harvested plants were dried at 35 °C for 7 days. The total dry biomass and inflorescences of each plant were weighed separately. Inflorescence samples from the field test plants were accidentally discarded, thus, chemotype data from field-tested plants are not available. The biomass and inflorescence weights of the diploids and triploids in the field tests were measured and compared by a two-sample t-test. Tetraploid plants were not assessed in the field trial due to the unavailability of seeds at the time of field trial establishment.

### 2.5. Flow Cytometry

The ploidies of all colchicine-treated plants, hybrid, and inbred plants that were used in this study were estimated by flow cytometry (Quantum P UV-LED, QuantaCyte, Mullica Hill, NJ, USA). For each plant, a 100–200 mg segment of petiole or fully developed leaf tissues were used. For small seedlings, a fully developed leaf was preferred, whereas for clonally propagated adult and colchicine-treated plants, petiole tissues were used. Tissue samples were cut into small (<1 mm^2^) pieces using a razor blade in 200 μL of nuclei extraction buffer solution (CyStain UV Precise P Nuclei Extraction Buffer; Sysmex, Görlitz, Germany). The chopped sample with buffer was filtered using a 30 μm gauge filter (CellTrics^®^, Münster, Germany), and the filtrate was collected in a 3.5 mL plastic tube (Sarstedt Ag & Co.; Nümbrecht, Germany). Ten to 30 μL of QA reference beads (UV bright 3 μm, Quantum Analysis GmbH, Münster, Germany) were mixed into the filtered nuclei buffer and were used as an internal standard. The peak of the internal standard was approximately the size of a triploid genome; thus, the internal standard was used only for identifying the diploid and tetraploid plants. For identifying the triploid plants, plant tissues of a known diploid were used as a standard. Relative fluorescence of total DNA content was then analyzed using the flow cytometer. Histograms of DNA content were created and visualized using CyPAD version 1.3 (Quantum Analysis GmbH, Münster, Germany). Three replicate flow cytometric results were used to define the ploidy of each hybrid plant that was used in fertility and phenotype tests.

### 2.6. Chromosome Squashes

Chromosome squashes were performed on three randomly chosen hybrid plants of each ploidy from the F_1_ hybrid plants. The method of root pre-treatment was modified using a protocol from Chen et al. [28]. Fresh root tips were collected and submerged in a solution made up of 2 mM 8-hydroxyquinoline and 0.24 mM cycloheximide at 4 °C for 6 h. Samples were then transferred into Farmer’s solution (3:1 ethanol to acetic acid by volume) and held overnight in a 25 mL glass bottle. The following day, root tips were transferred into 70% ethanol for long-term storage in a 4 °C refrigerator. An improved method for root preparation [29] with a 10-h enzyme digestion treatment was used. Following enzyme digestion, two drops of a modified Farmer’s solution (3:1 methanol to acetic acid by volume) were applied to the center of a glass slide, and macerated root tip cells were dispersed by lightly tapping with a metal spatula. Four drops of modified Farmer’s solution were then added to each corner of the slide before igniting the slide by passing it briefly through an alcohol lamp. Slides were allowed to dry in a 37 °C oven overnight. Dried slides were submerged in a 50× diluted Giemsa stain (Sigma-Aldrich, Saint Louis, MO, USA) for 5 min, rinsed in water for 30 s, and allowed to dry at 37 °C in an oven. Stained slides were screened for condensed chromosomes at a magnification of 4000× on a compound light microscope (Revolve, ECHO, San Diego, CA, USA).

## 3. Results

### 3.1. Ploidy Estimation

**Figure 2:**
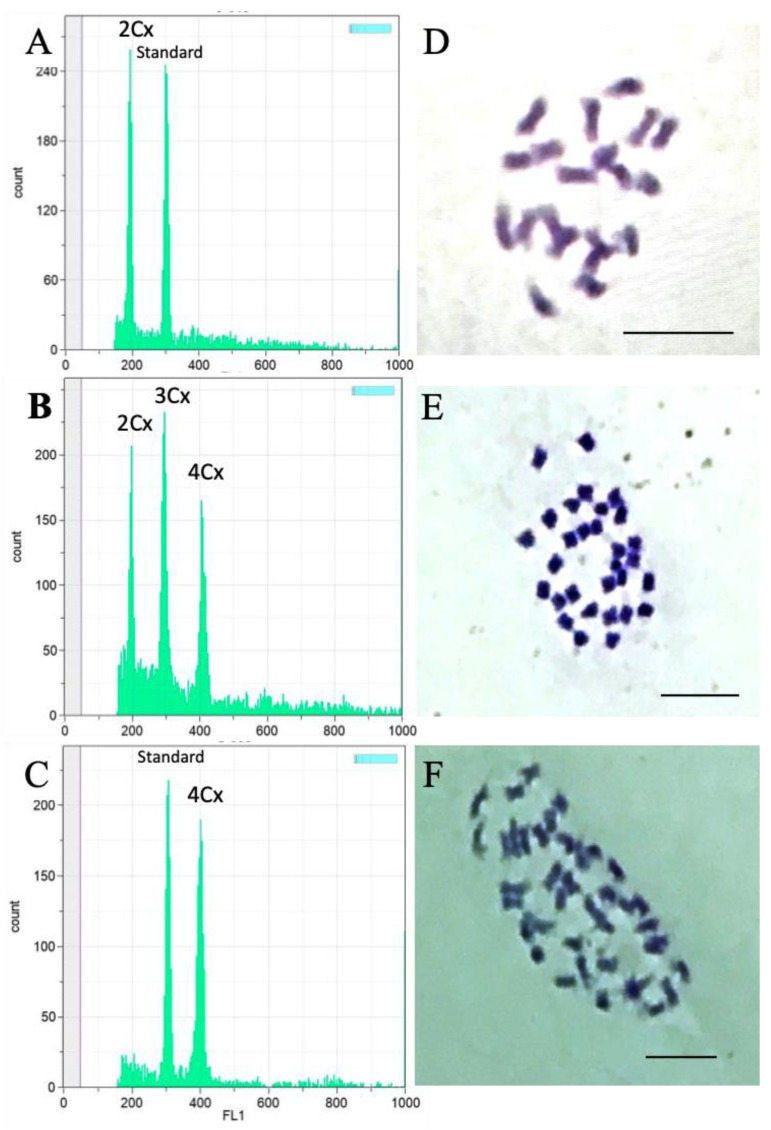
Ploidy identification by flow cytometry and chromosome squashes. Flow cytometric analysis of (**A**) a diploid *C. sativa* ‘Stem Cell CBG’ shown with the internal standard, (**B**) diploid, triploid, and tetraploid F_1_ hybrids, and (**C**) a tetraploid F_1_ hybrid and internal standard. Chromosome squash of (**D**) a diploid *C. sativa* ‘Stem Cell CBG’ (2n = 2*x* = 20), (**E**) a triploid ‘Stem Cell CBG Seedless’ (2n = 3*x* = 30), and (**F**) a tetraploid F_1_ hybrid (2n = 4*x* = 40). Internal standard (standard) = QA reference beads UV bright 3 μm (Quantum Analysis GmbH, Münster, Germany). Bar = 10 µm.

The ploidies of the parents and F_1_ hybrids were estimated using flow cytometry and chromosome squashes. Representative histograms from the flow cytometric analyses and images from the chromosome squashes are shown in Figure 2. Only plants with estimated ploidies were utilized in all the experiments.

### 3.2. Fertility and Crossing Compatibility

**Figure 3:**
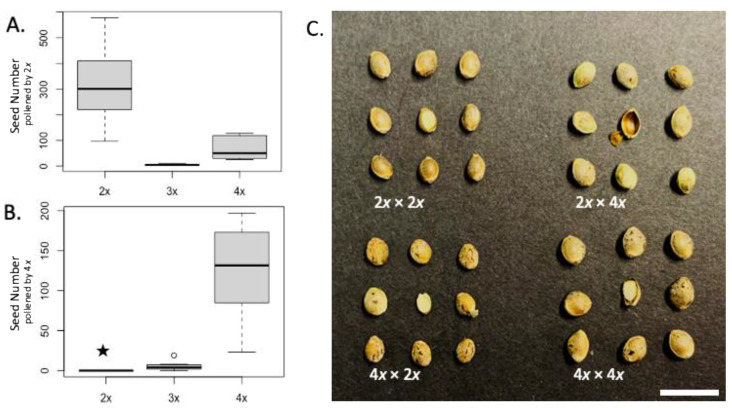
Results from interploidy crossing compatibility investigations of diploid, triploid, and tetraploid F_1_ hybrid *Cannabis sativa*. (**A**) Average seed count of the three ploidies of F_1_ hybrids, derived from crosses using diploid pollen donors and (**B**) tetraploid pollen donors. The star indicates that an average of 232.75 unfilled seeds and 0 filled seeds per plant were observed and the unfilled circle represents an observation outside of the 1.5 × inner quartile range cutoff as represented by the box and whiskers plots. (**C**) Seeds from several interploidy crossing combinations; one cut seed is displayed in the center of the grid to show seed development. Pericarps that developed as a result of the 2*x* × 4*x* crosses did not contain an embryo or endosperm. Bar = 1 cm.

Controlled pollination experiments, including crossing combinations using diploid, triploid, and tetraploid pollen recipients, and diploid and tetraploid pollen donors, demonstrated that diploid × diploid and tetraploid × tetraploid crosses were the most fertile. Fertility was defined in this experiment based on the average seed count and the number of viable seeds from each combination of pollinators. Seed viability was categorized as either “filled” (containing endosperm and an embryo), or “empty” or “abnormal” (containing a fully formed pericarp, but lacking endosperm or embryo) (see Figure 3). Tetraploid plants displayed reduced fertility with both the diploid and tetraploid pollen donors (Figure 3, Table 2). The average seed counts for the diploid × diploid, triploid × diploid, and tetraploid × diploid crossing combinations were significantly different at 317.25, 4.25, and 68.62 filled seeds per plant, respectively. In crosses with the tetraploid pollen donor, the average filled seed counts per diploid, triploid, and tetraploid plant were 0, 5.63, and 124.75, respectively (Figure 3, Table 2).

An individual plant was considered female infertile when all the crosses using that parent resulted in at least 95% reduced viable seeds as compared to a fertile female diploid plant. In other words, the average seed counts of all the crosses were compared to the average seed counts of the diploid × diploid cross, which was used as a standard. In our trials, using the triploid as the seed parent in crosses with the diploids and tetraploids resulted in an average of 4.25 and 5.63 filled seeds per cross, respectively, representing 1.3% and 1.8% of the seeds produced by the diploid × diploid cross. The triploids were therefore rated as “infertile”.

Notably, although the diploid × tetraploid crosses did not produce seeds containing endosperm or embryos, this crossing combination yielded, on average, 232.75 empty seeds with fully formed pericarps (Figure 3 and Table 2). The empty seeds appeared similar in size to those produced as a result of the tetraploid × diploid cross; however, the empty seeds were light in weight as compared with the filled seeds (Table 2), and could be easily broken. All of the seeds (pericarps) that formed as a result of the diploid × tetraploid cross lacked endosperm; the crosses involving triploids also had high rates of empty seeds (>50%), whereas diploid × diploid, tetraploid × diploid, and tetraploid × tetraploid crosses had less than 10% aborted seeds (Table 3). While most aborted seeds were smaller and abnormally shaped (Figure 3), the empty seeds from the diploid × tetraploid cross appeared visually normal.

Tetraploids displayed reduced female fertility as compared to diploids, but were still fertile. When pollinated by diploid plants, the tetraploid seed parents showed an average of 78% fewer filled seeds than the diploid × diploid cross (Table 2). A similar phenomenon was observed when using the tetraploids as pollen donors. When pollinated by tetraploid plants, the tetraploid seed parents showed 42% fewer total seeds than the diploid × tetraploid combination. These results, taken together, indicate that the tetraploid plants assessed in this study have reduced female fertility, but are still able to produce viable seeds.

Hybridizations between the diploids and tetraploids showed asymmetric crossing compatibility (Figure 3 and Table 2). Filled seeds were observed in the diploid x diploid, tetraploid × diploid, and tetraploid × tetraploid crosses (Table 2); however, no filled seed was developed as a result of the diploid × tetraploid cross. Although no fully developed seed was formed in the diploid × tetraploid cross, large numbers of empty seeds with fully developed pericarps were observed (Figure 3, Table 2).

Seed weights and sizes were impacted by both the seed parent and pollen donor. The average one hundred seed weights of the diploid × diploid, diploid × tetraploid, tetraploid × diploid, and tetraploid × tetraploid crosses were 1.25 g, 0.37 g, 1.3 g, and 2.07 g, respectively (Table 2). The seeds from the diploid × tetraploid cross were lighter in weight than those from the other crosses because the seeds lacked endosperm; the seeds from the tetraploid × tetraploid crosses were the largest and heaviest (Figure 3 and Table 2).

### 3.3. Biomass and Inflorescence Weights and Plant Architecture

Higher average total biomass and inflorescence weights were observed in triploid plants as compared to diploid plants in the field (Table 3). The average total plant biomass of the diploids and triploids in the field test were 2097.4 g and 3311.3 g, respectively. The average inflorescence weights of the diploids and triploids in the field tests were 1068.3 g and 1212.1 g, respectively. In the field, the triploids had an average 57% increase in biomass (*p*-value < 0.05) and 23% increase in inflorescence weight (*p*-value < 0.1) as compared to the diploids (Table 3). The average biomass of the diploid, triploid, and tetraploid in the indoor tests were 57.7 g, 67.8 g, and 58.2 g, respectively. Although these numbers appear different, statistical significance was not achieved. The average inflorescence weights, however, significantly differed among ploidies in the indoor trials. Triploids had statistically higher average inflorescence weights per plant (30.7 g) as compared with the diploids (24.3 g) and tetraploids (23.1 g) (Table 3).

**Figure 4:**
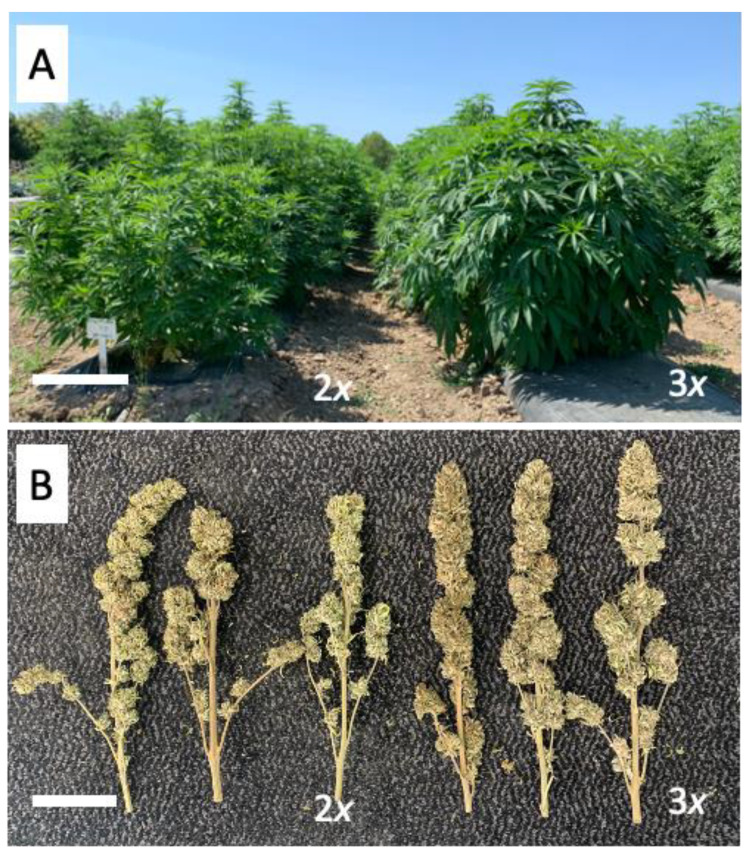
Whole-plant and inflorescence architectures of diploid and triploid hybrid *C. sativa* plants in the field. (**A**) Plants 60 days following transplant growing in the field. Diploid plants are on the left and triploid plants are on the right. Bar = 30 cm. (**B**) Dried terminal inflorescences of three diploid (left) and three triploid *C. sativa* plants (right). Bar = 10 cm.

The plant and inflorescence architectures were visibly different between the diploids and triploids in the field. Compared to the diploid plants, the triploid plants appeared taller and wider (Figure 4A). The leaves, shoots, and branches of the triploid plants appeared bigger than the diploid plants in the field (Figure 4). The inflorescence architecture of the triploid plants showed a visibly longer and more condensed, continuous inflorescence than the diploid plants, with fewer small branches (Figure 4B). Although visual observations were made on the differences in plant phenotype, no measurements were made.

### 3.4. Cannabinoid Yield

Significant differences in CBG(A) concentration and total cannabinoid concentration were observed between the diploids and tetraploids in the indoor trials. Although the triploid plants showed the highest total cannabinoid yield and CBG(A) yield per plant, the difference was not statistically different from either the tetraploids or diploids (Table 3). The total CBG(A) concentrations of the diploids, triploids, and tetraploids were 7.78%, 9.19%, and 11.23%, and the total cannabinoid concentrations were 8.66%, 10.18%, and 12.38%, respectively. Although there were differences in CBG(A) concentration among ploidies, the average THC(A) concentration was statistically the same (Table 3). The average total THC(A) concentrations of the diploid, triploid, and tetraploid plants were 0.141%, 0.138%, and 0.137%, respectively. The average cannabinoid yields, estimated by total CBG(A) concentration × inflorescence weight of untrimmed inflorescences, of the diploid, triploid, and tetraploid plants, were 2.01 g, 3.11 g, and 2.93 g, respectively; the differences in yield were not statistically significant (Table 3).

## 4. Discussion

### 4.1. Fertility, Infertility, and Crossing Compatibility

Based on the reduction in seed development as compared to other crossing combinations, the triploids were determined to be infertile. Infertile hemp has been recommended as a strategy to mitigate yield reduction caused by pollination [13]. As far as we are aware, this research represents the first report of triploid *C. sativa* female infertility. Whether the infertility of the triploids directly mitigates the yield reduction by pollination was not addressed in this study, and therefore is still unknown. Further research is necessary to demonstrate the effect of infertility on the development of cannabinoids in high pollen versus low or no pollen conditions.

Nonetheless, it can be speculated that infertility might improve cannabinoid yield by the following two different mechanisms: by avoiding the termination of inflorescence development and/or by avoiding reductions in cannabinoid accumulation. Similarly to *Arabidopsis*, *C. sativa* has an indeterminate inflorescence architecture, which means the inflorescences keep growing and developing additional flowers until a specific event sends a signal to halt the flowering process [30]. In the *Arabidopsis* model, successful pollination can induce AGAMOUS expression [31,32], which will trigger downstream signals to end flowering and begin flower senescence [30]. The triploid plants in our research showed no response to pollination, which could be a sign that the terminal flowering signals might not initiate in the triploid plants following a pollen challenge. On the other hand, seed development after pollination may alter carbon resource allocation, which might reduce the development of inflorescences and secondary metabolism synthesis. Our research showed that triploid plants rarely produced seeds following pollination, and could therefore suggest that plants will not allocate carbon resources from flower development or cannabinoid synthesis to seed development. It is also possible that neither of these physiological processes are impacted, but rather the reduction in cannabinoid content in pollinated (and seeded) flowers is merely due to a dilution effect of the presence of seeds, which do not contain the cannabinoid-producing trichomes. More research on the effect of infertility on cannabinoid development is warranted to further confirm the value of commercial triploid cultivars.

Although plants containing even numbers of chromosomes are generally considered fertile, reduced fertility or even infertility have been reported in other species containing even ploidies. For example, the allotetraploid, or natural tetraploid, *Hibiscus acetosella* ‘Panama Red’ has been reported as producing no viable seeds [33]. Tetraploid foxtail millet (*Setaria italica*) cultivars displayed a two-fold reduction in fertility as compared to their diploid counterparts [34]. Reporting the reduced fertility of tetraploid *C. sativa* has important implications for the hemp breeding and hemp seed industries. Our results indicate that the investment of producing triploid seeds may be higher than producing diploid seeds, due to the lower seed numbers produced in the tetraploid × diploid crosses studied in this research. To compensate for the reduced seed number, more intense pollination or extending the pollination period may be recommended. Furthermore, the asymmetric nature of the crosses involving the tetraploid, at least as observed in the genotypes tested in the current study, indicate that the selection and breeding of the pollen recipient as the tetraploid parent has important ramifications for the success in breeding triploid *C. sativa*.

The unidirectional compatibility or asymmetric interploidy crossing compatibility of hemp might be caused by an asymmetric triploid block. A triploid block is a phenomenon in which seed development fails due to an imbalance in genome size and gene expression between the parents of different ploidies; in many cases, triploid block leads to abnormal development or underdevelopment of the endosperm and embryo [35]. Studies in *Brassica oleracea* showed the same asymmetrical interploidy compatibility pattern as we observed in *C. sativa*; that is, when the paternal parent had the higher chromosome number, there was a lethal disruption in embryo development, whereas when the maternal parent contained a chromosome excess, viable seeds were formed [36]. Imbalances in the expression levels of the AGAMOUS-like gene families appeared to play important roles in the endosperm and embryo development failure in *B. oleracea* [36]. Research on potato showed that the strength of triploid block can vary among genotypes [35]. Therefore, research into multiple genotypes and gene expression variations may be useful in obtaining a full understanding of asymmetrical compatibility and asymmetric triploid blocking in *C. sativa* as a species.

### 4.2. Cannabinoid and Biomass Yield

This study showed the potential for ploidy manipulation in the improvement of hemp cultivar biomass and cannabinoid yield. The triploid CBG-dominant F_1_ hybrid plants showed higher biomass, inflorescence weights, and cannabinoid concentrations as compared to the diploid plants. Although the differences in cannabinoid concentrations and total CBG(A) concentration were not statistically significant between the diploids and triploids, the approximately 1.5% increase in each may be of economic importance to growers.

Notably, the total THC(A) concentration did not increase with the total CBG(A) concentration and ploidy. These results mimic those shown in other studies on the ploidy manipulation of *C. sativa* [23,24]. These results are valuable to breeders and growers interested in triploid *C. sativa* cultivars, given the current strict limitations on THC(A) content in industrial hemp plants and plant products. US federal regulations currently limit industrial hemp products to a total THC(A) concentration of 0.3%; therefore, it is important that increases in dominant cannabinoid content do not simultaneously push the total THC(A) content over this threshold. Future studies involving larger population sizes, more genotypes, *C. sativa* containing other dominant cannabinoids such as CBD or THC, and those investigating the effects of environmental factors on polypoid performance would help elucidate any additional benefits of ploidy manipulation in *C. sativa* for cultivar improvement.

### 4.3. Future Directions in Hemp Polyploid Manipulations

Several methods of ploidy manipulation in *C. sativa* were published prior to this study; however, this is the first report that tracks phenotypic differences between diploids, triploids, and tetraploids in relation to seed production, biomass, and cannabinoid yield. We also further report the crossing compatibility between these ploidies. Although several novel findings have been reported, additional research on hemp ploidy manipulation is warranted to fill in the gaps left by this study. The plants used in this study were from a single genotype, and in the case of the clonally propagated TS1-3, only one colchicine-treated individual was used in the trials. Off-target mutations not identified by flow cytometry, including smaller (non-chromosome level) deletions or insertions, or other effects independent of genome doubling, are known to occur as a result of colchicine treatments [37]. For example, in poinsettia, various morphological mutations were observed following treatment with colchicine [17]. In *Arabidopsis*, colchicine treatment also resulted in plant performance differences in their progenies [37]. Therefore, repeating these trials with several different colchicine-treated individuals of the same genotype would help elucidate the reproducibility of the results we report herein. Ploidy manipulations, using both colchicine [13,23] and oryzalin [24], have been reported in *C. sativa*; however, the non-ploidy mutation effects of these chemicals remain unknown. Further research on specific chemicals and concentrations, and their effects on off-target mutations in the hemp genome, would be beneficial for the breeding of polyploid *Cannabis*.

Additional research investigating the effect of polyploidy in *Cannabis* breeding, which includes several additional genotypes and reciprocal crosses, would also be valuable in understanding the applicability of the results described in this study to the species as a whole. In the present research, we only included one genotype of an F_1_ hybrid triploid from a single directional cross, due to limitations in pollen viability. Research that includes multiple genotypes is needed to understand the interactions between ploidy and genotype. Comparisons using diploids and triploids, each produced from reciprocal crosses, will help elucidate quantitative trait improvements between specific parental gene doses and the effects of polyploidy. The effect of chromosome doubling in the parents would also be valuable in future studies to help detangle the effects of hybridization versus polyploidy.

A final future area of investigation may be into the use of true male pollen in testing the sterility of the triploid *C. sativa* plants. Previous research has indicated that the pollen from reversed female *C. sativa* plants is frequently less viable than the pollen from true genetic males [38]. Since pollen from true males is a contaminant problem from fiber and grain crops, these additional experiments are warranted to ensure that triploid plants will maintain their sterility in field conditions. Understanding the dosage and timing of pollination may also be valuable to confirming the value of using triploids in essential oil crops.

## Figures and Tables

**Table 1:** Plant material and crossing combinations used in trials.

Tests	Ploidy	Plant Material ^1^	Number of Plants
**Fertility**	2*x*	F_1_ hybrid, TS1-3 (2*x*) × P163 (2*x*) ^2^	8
	3*x*	F_1_ hybrid, TS1-3 (4*x*) × P163 (2*x*) ^3^	8
	4*x*	F_1_ hybrid, TS1-3 (4*x*) × P163 (4*x*)	8
	2*x*	F_1_ hybrid, TS1-3 (2*x*) × P163 (2*x*) ^2^	8
	3*x*	F_1_ hybrid, TS1-3 (4*x*) × P163 (2*x*)	8
	4*x*	F_1_ hybrid, TS1-3 (4*x*) × P163 (4*x*)	8
**Indoor test**	2*x*	F_1_ hybrid, TS1-3 (2*x*) × P163 (2*x*) ^2^	7
	3*x*	F_1_ hybrid, TS1-3 (4*x*) × P163 (2*x*) ^3^	7
	4*x*	F_1_ hybrid, TS1-3 (4*x*) × P163 (4*x*)	7
**Field test**	2*x*	F_1_ hybrid, TS1-3 (2*x*) × P163 (2*x*) ^2^	15
	3*x*	F_1_ hybrid, TS1-3 (4*x*) × P163 (2*x*) ^3^	15

^1^ ‘TS1-3’ and ‘P163’ are female inbred lines developed by Oregon CBD. ^2^ This cross is sold commercially as ‘Stem Cell CBG’. ^3^ This cross is sold commercially as ‘Stem Cell CBG Seedless’.

**Table 2:** Average seed number, seed weight, and crossing compatibility of intra- and inter-ploid hybridizations.

Seed Parent	Pollen Donor	Filled Seed Number ^1,2^	Empty or Abnormal Seed Number	Total Seed Number	Compatibility ^4^	Weight of 100 Seeds (g) ^3^
2*x* F_1_ hybrid	2*x* F_1_ hybrid	317.25 ^a^	36.75	354	Yes	1.25 ^b^
3*x* F_1_ hybrid	2*x* F_1_ hybrid	4.25 ^b^	4	8.25	-	-
4*x* F_1_ hybrid	2*x* F_1_ hybrid	68.63 ^c^	17.25	99.75	Yes	1.30 ^b^
2*x* F_1_ hybrid	4*x* F_1_ hybrid	0 ^A^	232.75	232.75	No	0.37 ^d^
3*x* F_1_ hybrid	4*x* F_1_ hybrid	5.63 ^B^	11.63	17.25	-	-
4*x* F_1_ hybrid	4*x* F_1_ hybrid	124.75 ^C^	10.13	134.88	Yes	2.07 ^a^

^1^ Means separations were conducted among ploidies pollinated by the same pollen donor; therefore, means separations in upper case and lower case represent separate post hoc comparisons. ^2^ Means followed by the same letter are not statistically different. For all statistical analyses, an initial analysis of variance was performed followed by a Tukey’s means separation test using a 99% confidence interval. ^3^ One hundred seed weights were based on 100 seeds (three replicated measurements), which were fully developed in size and shape (filled and unfilled seed were randomly selected). Although the 2*x* × 4*x* cross did not result in filled seed, the weight of seeds that had a fully developed pericarp, but lacked endosperm or an embryo, is reported. A dash (-) indicates that too few seeds were produced to calculate an average seed weight. ^4^ Compatibility was defined as the ability for parents known to be fertile to make seeds in the cross specified. Compatibility was not assessed for triploids (indicated by a dash [-]) as triploids were determined to be infertile as a seed parent.

**Table 3:** Phenotypic traits of intra- and inter-ploid hybrids of *C. sativa*.

Female Material	Number of Plants	Biomass Per Plant (g) ^1^	Inflorescence Weight Per Plant (g) ^1^	Total CBG Concentration (%) ^1^	Total THC Concentration (%) ^1^	Total Cannabinoid Concentration (%) ^1^	CBG Yield Per Plant (g)	CBG: THC
2*x* F_1_ hybrid-field	15	2097.4 ^A^	1068.3 ^A^	-	-	-	-	-
3*x* F_1_ hybrid-field	15	3311.3 ^B^	1312.1 ^B^	-	-	-	-	-
2*x* F_1_ hybrid-indoor	7	57.7 ^a^	24.3 ^a^	7.78 ^a^	0.141 ^a^	8.66 ^a^	2.01 ^a^	64:1
3*x* F_1_ hybrid-indoor	7	67.8 ^a^	30.7 ^b^	9.15 ^ab^	0.138 ^a^	10.18 ^ab^	3.11 ^a^	73:1
4*x* F_1_ hybrid-indoor	7	58.0 ^a^	23.1 ^a^	11.23 ^b^	0.137 ^a^	12.38 ^b^	2.93 ^a^	84:1

^1^ Pairwise means separations were conducted among plants grown within either the field or indoor trials; therefore, means separations in upper case and lower case represent separate post hoc comparisons. Means followed by the same letter are not statistically different. Statistical analyses of cannabinoids were conducted using a Kruskal–Wallis H test and pairwise Wilcoxon rank sum tests with a 90% confidence interval. Biomass and inflorescence weights were compared using a two-sample *t*-test.
